# Multiplex lateral flow assay development for snake venom detection in biological matrices

**DOI:** 10.1038/s41598-024-51971-2

**Published:** 2024-01-31

**Authors:** Cecilie Knudsen, Selma B. Belfakir, Pelle Degnegaard, Jonas A. Jürgensen, Aleksander M. Haack, Rasmus U. W. Friis, Søren H. Dam, Andreas H. Laustsen, Georgina M. S. Ross

**Affiliations:** 1 VenomAid Diagnostics, 2800 Kongens Lyngby, Denmark; 2 Department of Biotechnology and Biomedicine, Technical University of Denmark, 2800 Kongens Lyngby, Denmark

**Keywords:** Assay systems, Antibody generation, Diagnostic markers

## Abstract

*Bothrops* and *Lachesis* are two of Brazil’s medically most relevant snake genera, causing tens of thousands of bites annually. Fortunately, Brazil has good accessibility to high-quality antivenoms at the genus and inter-genus level, enabling the treatment of many of these envenomings. However, the optimal use of these treatments requires that the snake species responsible for the bite is determined. Currently, physicians use a syndromic approach to diagnose snakebite, which can be difficult for medical personnel with limited training in clinical snakebite management. In this work, we have developed a novel monoclonal antibody-based multiplex lateral flow assay for differentiating *Bothrops* and *Lachesis* venoms within 15 min. The test can be read by the naked eye or (semi)-quantitatively by a smartphone supported by a 3D-printed attachment for controlling lighting conditions. The LFA can detect *Bothrops* and *Lachesis* venoms in spiked plasma and urine matrices at concentrations spanning six orders of magnitude. The LFA has detection limits of 10–50 ng/mL in spiked plasma and urine, and 50–500 ng/mL in spiked sera, for *B. atrox* and *L. muta* venoms. This test could potentially support medical personnel in correctly diagnosing snakebite envenomings at the point-of-care in Brazil, which may help improve patient outcomes and save lives.

## Introduction

Annually, approximately 27,000 people are bitten by venomous snakes in Brazil, resulting in many fatalities and even more permanent morbidities that deteriorate the victims’ quality of life^1–4^. The only specific treatment for snakebite envenoming is antivenom. In Brazil, antivenoms are available at the genus and inter-genus levels. Antivenoms are most effective against envenoming from snake species whose venom was used in their production, but usually, they can neutralize closely related venoms. Therefore, it is essential to determine whether patients are envenomed and, if so, which antivenom to administer^1^. Currently, snakebite envenoming is diagnosed by clinical personnel via a syndromic approach, by patient history, witness statements, and occasionally identification of dead or photographed snakes^5^. Unfortunately, not all medical staff have the specialized knowledge required to diagnose snakebite^6^, which could be problematic if it delays treatment^7,8^.

Two of the most medically relevant Brazilian snake genera are *Bothrops* (67%) and *Lachesis* (22%), responsible for 89% of all identified snakebites in Amazonas, Brazil^9^. Both groups of vipers possess venom with coagulopathic, proteolytic, and hemorrhagic effects^5^. The overlap in clinical manifestations following envenoming makes it challenging to distinguish them using a syndromic approach alone. In fact, a study of more than 5000 patients envenomed by *Lachesis spp.* in the Brazilian Amazon found that 12.8% of the patients received antivenoms not indicated for *Lachesis* bites^10^, potentially indicating misdiagnoses. Misdiagnosis of *Lachesis* bites is particularly problematic as victims require polyvalent bothro-lachetic antivenom. In contrast, those bitten by *Bothrops* can receive either the polyvalent antivenom(s) or the monovalent bothropic antivenom^10^.

Analytical tools that can rapidly detect venoms could support healthcare workers in administering an appropriate antivenom. One way to achieve this is by developing multiplex tests that can differentiate venoms in biological samples. Multiplexing can improve user-friendliness, reduce costs, and save time, as only one test is required to detect multiple targets. Multiplex tests are especially relevant when samples are limited and where rapid decision-making is needed. However, optimizing multiplex tests is complex; avoiding cross-reactivity between the different binders and targets and non-specific binding with matrices is critical.

Several immuno- and DNA-based multiplex methods have emerged for snake venom detection^5,11^, including enzyme-linked immunosorbent assays (ELISAs)^12^, aptamer-linked immobilized sorbent assay (ALISA)^13^, agglutination assays^14^, optical immunoassays^15^, fluorescent sensor microarrays^16^, polymerase chain reactions^17^, loop-mediated isothermal amplifications^18^, impedimetric immunosensors^19^, and mass spectrometry^20^. However, these proof-of-concept methods are laboratory-based and require extensive time (> 2 h), dedicated equipment, and trained personnel to carry out. One multiplex immunoassay kit is available commercially but only for the Australian market^21^, leaving geographical regions with more significant snakebite burdens uncovered. The World Health Organization (WHO) recognizes that snakebite diagnostics have the potential to inform the correct selection of antivenoms to treat patients by differentiating the most likely genus of snake responsible for the bite^22^. Yet, currently, no guidelines have been published for developing such tools.

Attention has shifted toward developing more accessible tools for multiplex venom detection at the point-of-care (PoC), such as lateral flow assays (LFAs). In 2006, the WHO introduced the ASSURED criteria for developing PoC diagnostics for sexually transmitted infections: affordable, sensitive, specific, user-friendly, rapid, robust, equipment-free, and deliverable to end users^23^. Recently, these criteria have been expanded to (RE)ASSURED by including *real-time connectivity* and *ease of specimen collection*. Digital readers, such as smartphones, enable LFA results to be recorded and transmitted in *real-time* or processed (offline) directly on the device^24^. Likewise, smartphone-based readouts can minimize misinterpretation of ambiguous results by end users. Meeting the (RE)ASSURED criteria remains challenging, especially considering the collection of invasive specimens, such as blood, plasma, and serum^25^. Snakebite diagnostics should be developed to work with non-invasive samples, such as urine, sweat, saliva, or wound swab samples. Still, sample collection and preparation can influence the matrix, which is vital to consider because assay performance is matrix-dependent^26^.

LFAs, which can quickly and qualitatively differentiate between bites, allowing more specific antivenoms to be used, are necessary^27^. Such a PoC test could improve doctors’ confidence in identifying the perpetrating snake genus rather than relying on their judgement^27,28^. In turn, this could prevent wasteful administration of inappropriate antivenoms, which are typically in short supply^29^. At the same time, a simple-to-use diagnostic tool could help snakebite victims to quickly seek hospital treatment rather than resorting to traditional healers. Both singleplex and multiplex snake venom detection LFAs have been developed, as highlighted in recent reviews^5,11^, and summarized in Table 1. However, these LFAs cannot be appropriately compared with each other as they each have distinct assay protocols, including different running buffers, sample dilution factors, and readout methods. Moreover, the LFAs do not target the same snake species and can use either polyclonal antibodies (pAbs) purified from antivenoms that target the whole venom or monoclonal antibodies (mAbs) developed to target a specific venom toxin. Likewise, it is difficult to benchmark the analytical performance of these LFAs as no standard methods or certified reference materials exist for snake venoms and the current ‘gold standard’ for diagnosing envenomation relies on a syndromic approach and the victim bringing the perpetrating snake with them to the hospital.

**Table 1:** Summary of existing proof-of-concept snake venom detection LFAs.

Singleplex/multiplex	Target spp.	Target area	Antibodies	NPs	Test duration	Limit of Detection^a^	Matrices tested	Refs.
Singleplex	*N. atra*	Asia	Duck & rabbit pAbs	AuNPs	20 min	5 ng/mL in serum	Serum from patients	^32^
Multiplex	*N. naja* *D. russelii*	Asia	Rabbit pAbs	AuNPs	5–10 min	0.1 ng/mL in buffer for *N. naja* & *D. russelii*	Plasma from envenomed mice	^33^
Multiplex	*T. stejnegeri* *P. mucrosquamatus B. multicinctus* *N. atra*	Asia	Horse pAbs	AuNPs	10 min	5 ng/mL in plasma for *B. multicinctus & N. atrox* and 50 ng/mL in plasma for *T. stejnegeri & P. mucrosquamatus*	Spiked human plasma & serum from patients	^12^
Singleplex	*Naja* spp.	Asia, Africa	Horse & rabbit pAbs	AuNPs	20 min	5–500 ng/mL in serum depending on which of eight species was tested	Spiked fetal bovine serum	^34^
Multiplex	*D. russelii*	Asia	Horse & goose pAbs	AuNPs	25 min	10 ng/mL in fetal bovine serum	Spiked fetal bovine serum & serum from patients	^35^
Singleplex	*N. naja* *B. caeruleus*	Asia	Mouse mAbs	AuNPs	Not reported	5000–250,000 ng/mL in buffer for recombinant toxin, 25,000–250,000 ng/mL in buffer for *N. naja*, 25,000–250,000 ng/mL in buffer for *B. caeruleus*	Spiked fetal bovine serum	^36^
Multiplex	*B. multicinctus* *N. atra*	Asia	Horse pAbs	AuNPs	10–15 min (enrichment) + 15 min (LFA)	5 ng/mL (without enrichment) and 1 ng/mL (with enrichment) in plasma for *B. multicinctus*. 5 ng/mL (with enrichment) in plasma for *N. atra*	Spiked human plasma	^31^
Singleplex	*B. multicinctus*	Asia	Rabbit pAbs	AuNPs	10–15 min	1 ng/mL in buffer for *B. multicinctus*	Tissue homogenates, blood, and urine from envenomed rats	^37^
Singleplex	*Bothrops *spp.	Latin America	Mouse mAbs	AuNPs	5 min (pre-incubation) + 15 min (LFA)	10.3 ng/mL in buffer, 9.5 ng/mL in urine, 8.0 ng/mL in serum by reader and 25 ng/mL in buffer by naked eye for *B. atrox*	Spiked human urine, serum, & plasma	^38^

NPs is short for nanoparticles, AuNPs for gold nanoparticles, pAbs denote polyclonal antibodies, and mAbs denote monoclonal antibodies.

^a^The reported detection limits are contingent on the various dilution factors used in these studies and should only be compared when considering these factors.

The LFAs reported in Table 1 have limits of detection (LoD), which mostly fall within a clinically relevant range of venom concentrations for the first 50 h post-snakebite (for Vipers: < 1–1000 ng/mL; for Elapids 0.1– > 1000 ng/mL)^30^. Yet, detection limits are impacted by biological matrices where biomolecules can interact with the analyte or the binders, influencing the assay sensitivity by introducing non-specific matrix interactions^26^. Recently, a method was developed for eliminating plasma matrix effects by pre-enriching venoms using a cation exchange tip, but this method only works for elapid venoms^31^.

A multiplex LFA prototype using two sets of antibody sandwich pairs was developed and optimized for detecting Brazilian *Bothrops* and *Lachesis* venoms in biological matrices within 15 min. The assay was validated by testing venom concentrations spanning six orders of magnitude in six different sample matrices to assess its strengths and limitations. The LFA signals were recorded using a smartphone with a 3D-printed attachment for subsequent (semi)quantitative analysis of the results. The LFA developed here may be applied to detect venom components from at least nine *Bothrops* spp. and one *Lachesis* spp. to support snakebite management in Brazil.

## Results and discussion

### Antibody development and characterization

The immunization and hybridoma generation resulted in 53 monoclonal cell lines. The *Lachesis* sandwich pair was selected for further experiments from the antibodies derived from these cell lines. In an indirect sandwich ELISA, the selected sandwich pair elicited signals indicative of binding to venom components from *L. muta* and *L. melanocephala*, but not towards *Bothrops* or *Crotalus* venoms, indicating that the antibodies were specific towards their target (Supplementary Information (SI) Fig. S1). Meanwhile, the selected *Bothrops* sandwich pair elicited strong signals on the venoms from several different *Bothrops* species and no signal on venoms from *Crotalus* or *Lachesis* species (SI Fig. S1). Therefore, this mAb pair was selected for application in the LFA as it enabled the specific detection of most of the tested Brazilian *Bothrops* spp. venoms without cross-reacting with venoms from other medically relevant snakes in the region.

### Evaluation of singleplex LFAs

The *Bothrops* LFA was tested in four blank matrices using running buffers with two different surfactants (*i.e.*, Tween-20 or Tergitol) with and without 4% NaCl. First, borate buffer with 1% BSA and 0.05% Tween-20 was tested as this running buffer has been successfully used in previous works with CNP-based LFAs without any false positives^39–41^. As can be seen from SI Fig. S2 and SI Table S1, all sample matrices tested in the NaCl-free running buffers had non-specific binding, resulting in false positives; the most intense test line was observed using the running buffer with Tergitol. Adding NaCl to the running buffer reduced the false positives in serum samples, indicating it could inhibit non-specific binding caused by matrix effects, as others have reported^31,42^. It is essential to consider the intended sample matrix early in the LFA development process, as success in buffer systems rarely reflects how the assay will perform in biological matrices^26^.

It was necessary to include salt in the running buffer to reduce the false positives for the *Bothrops* LFA. Therefore, buffer optimization experiments for the *Lachesis* LFA were performed with either Tween-20 (SI Fig. S3a) or Tergitol (SI Fig. S3b) and 4% NaCl in the buffer. Despite the presence of NaCl, both blank serum matrices resulted in the appearance of an intense test line for the *Lachesis* LFA indicating some matrix effect. In contrast, a false positive only occurred in the plasma matrices tested with the running buffer with Tergitol (SI Fig. S3b). Researchers have reported that testing sera can result in non-specific binding, possibly due to the presence of naturally occurring, poly-specific, and low-affinity heterophile antibodies^42,43^. The sample matrix (*i.e.,* serum) might require further dilution in the running buffer to reduce such non-specific binding. The combination of 4% NaCl and Tween-20 effectively reduced or eliminated the false positives, and therefore, these components were incorporated into the running buffer for further experiments. The inclusion of salt in the running buffer is a compromise between limiting the false positives and reducing the signal intensity on the test and control lines, which could decrease the overall assay sensitivity.

After optimizing the running buffer, the visual LoDs for the singleplex LFAs were determined. A concentration range of 1000–1 ng/mL was selected as it has been reported that this is a clinically relevant venom range for the first 50 h post viper-bite^30^. In both singleplex LFAs, there was an apparent decrease in test line signal intensity with decreasing venom concentration, with a visual LoD of 10 ng/mL and 50 ng/mL being reached with *B. atrox* and *L. muta* venoms respectively (SI Fig. S4).

### Comparison of multiplex LFA configurations

Two multiplex LFAs were developed with different test line configurations (*i.e.*, alternating the position of the upper test line between *Bothrops* and *Lachesis*) for comparison. In both configurations, the test line intensities and corresponding T/C ratios correlated with the tested venom concentrations.

The visual LoDs of configuration 1 (SI Fig. S5a) in spiked running buffer, were 10 ng/mL and 100 ng/mL in *B. atrox* and *L. muta* venoms, respectively. The signal on the *Bothrops* line was consistently more intense than the *Lachesis* line’s signal, resulting in a higher B/C compared with the L/C value at all tested concentrations. The same visual LoDs were reached with configuration 2 (SI Fig. S5b), but a weak signal appeared at the lower line (*Bothrops* line) between 10 and 1000 ng/mL in *L. muta* venom. Moreover, a weak signal also developed on the *Bothrops* line in blank running buffer which was indistinguishable from the signal at 1 ng/mL in *B. atrox* venom-spiked running buffer. The *Bothrops* line signal intensities were comparable between both configurations in *B. atrox* venom and in both venoms simultaneously. However, in configuration 2 the *Lachesis* line intensity (and corresponding L/C value) was reduced in *L. muta* venom and in both venoms.

Both configurations were tested in two blank serum and two blank plasma samples. Weak test line signals developed in the blank serum matrices in both multiplex configurations (SI Fig. S6), indicating that the matrix effect caused by serum could not be eliminated by diluting the matrix 10:90 in the running buffer. In contrast, a test line only developed in blank plasma citrate with configuration 2, demonstrating that this configuration was more susceptible to false positives. As a result, configuration 1 was selected for the remaining experiments.

**Figure 1:**
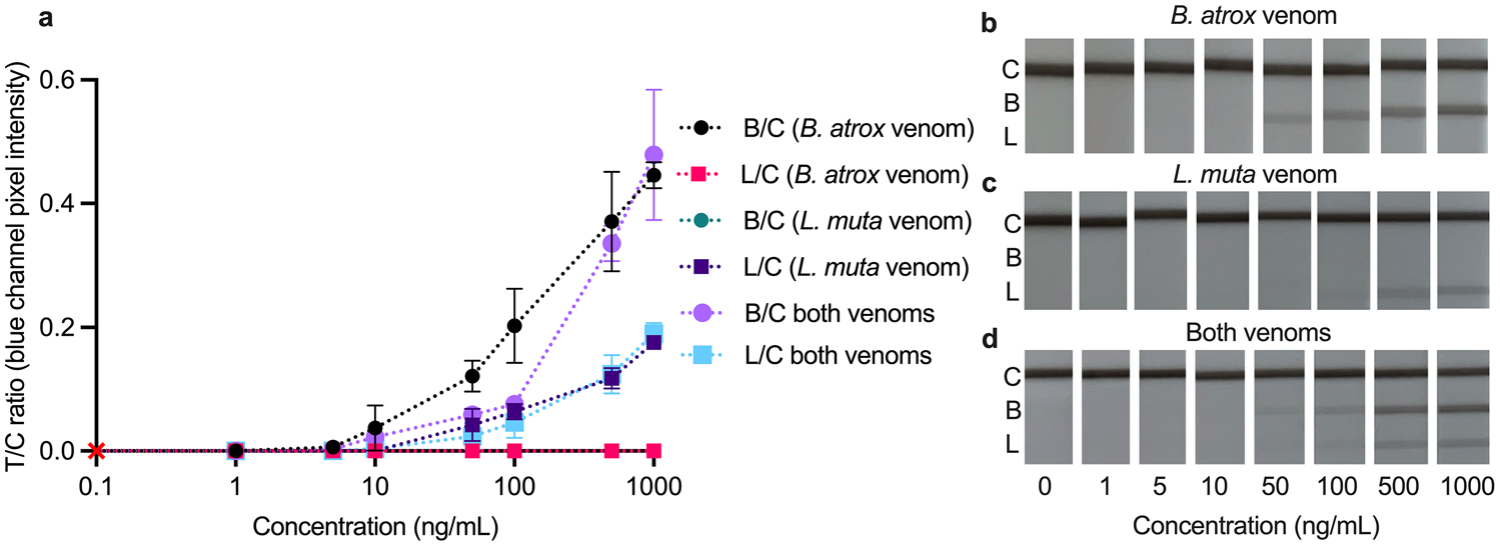
Calibration curves (**a**) and smartphone photos (**b**–**d**) of the multiplex LFA tested in (**b**) *B. atrox,* (**c**) *L. muta,* and (**d**) both venoms, spiked in increasing concentrations (1–1000 ng/mL) into the running buffer. Calibration curves plotted as test lines divided by control lines (T/C) ratios (*Bothrops*/control = B/C; *Lachesis*/control = L/C) against venom concentration. Error bars represent the standard deviation (n = 3), and the red cross represents the signal in blank running buffer.

An essential feature of multiplexing is distinguishing between or detecting more than one target in a single sample. In the larger concentration range (Fig. 1), the multiplex LFA had visual LoDs of 10 ng/mL for *B. atrox* (Fig. 1b), 50 ng/mL for *L. muta* (Fig. 1c), and 50 ng/mL for both venoms (Fig. 1d). However, because both mAb pairs used in the LFA target specific toxins within the venoms, not the whole venoms, the actual toxin concentration being detected is lower than reported here.

The purpose of this LFA is to facilitate the differentiation of snakebites caused by *Bothrops* and *Lachesis* which cause overlapping clinical manifestations in victims, rather than to detect the presence of both venoms simultaneously, so in the remaining experiments matrices were only spiked with a single venom rather than a cocktail of both.

### Cross-reactivity toward other venoms

**Figure 2:**
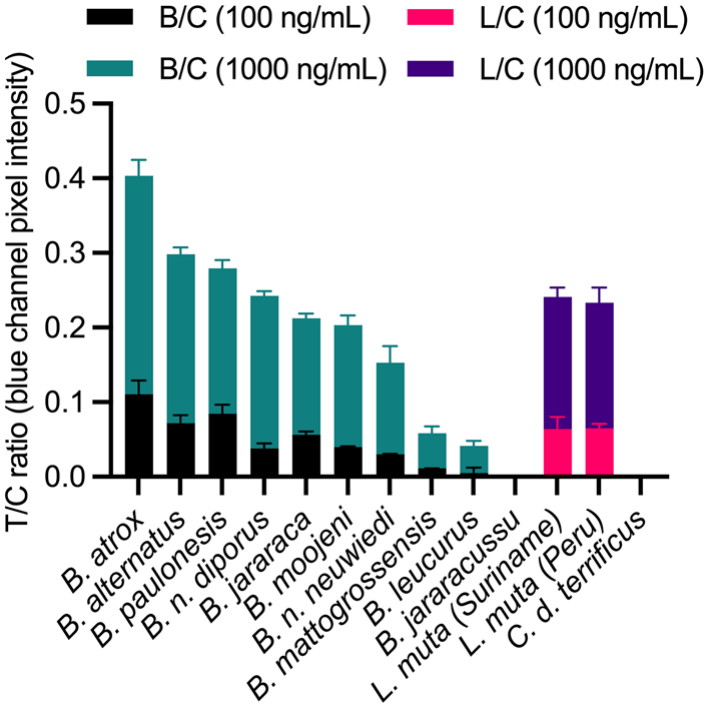
Detection of different target and non-target venoms spiked into running buffer plotted as test lines divided by control lines (T/C) ratios (*Bothrops*/control = B/C; *Lachesis*/control = L/C) against the different venoms at 100 and 1000 ng/mL Error bars represent the standard deviation (n = 2).

The LFA was tested in other relevant *Bothrops*, *Lachesis,* and *Crotalus* venoms from indigenous Brazilian species. However, it is important to note that some *Bothrops* and *Lachesis* spp. display ontogenetic and geographic venom variations^44–46^ and the results reported here relate to the venoms described in SI Table S2. The LFA could detect *Bothrops* and *Lachesis* venoms (Fig. 2), except for *B. jararacussu* and exhibited a concentration-dependency with consistently darker test lines at 1000 ng/mL compared to 100 ng/mL. Still, only two concentrations were tested, and the signal intensities were weak at 100 ng/mL in *B. mattogrossensis* and *B. leucurus* venoms indicating the LFA might not detect these venoms at lower concentrations. Importantly, no test line signal developed with *C. d. terrificus* venom at either concentration. It would be desirable to detect *C. d. terrificus* venom, but this would require an additional test line using a mAb pair specific toward *Crotalus*. Besides, bites caused by *Crotalus* result in clinical manifestations that are distinct from those caused by *Bothrops* or *Lachesis,* making it more straightforward to diagnose *Crotalus* envenoming by classical syndromic approaches^5^.

### Evaluation of multiplex LFA in spiked matrices

**Figure 3:**
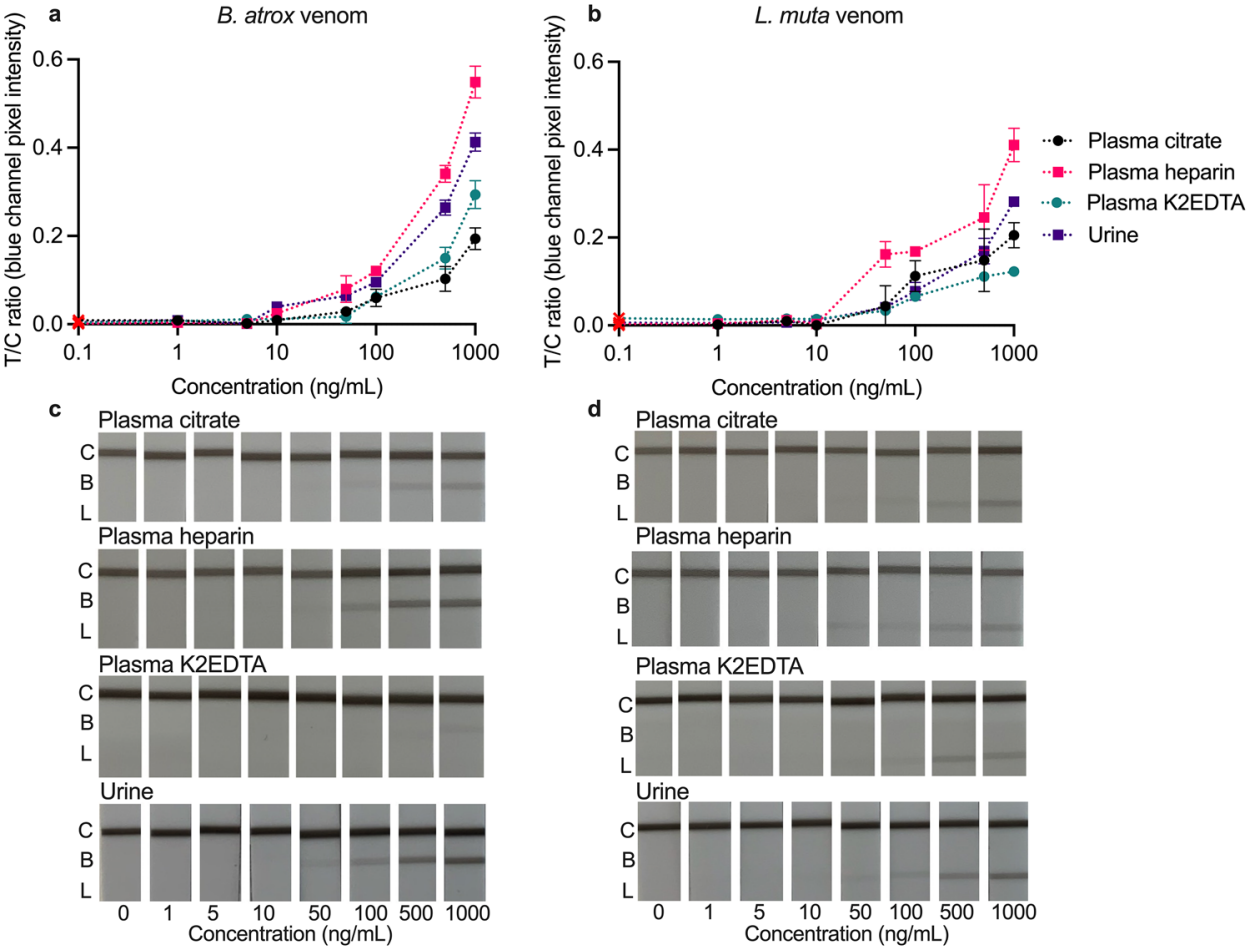
Calibration curves (**a**, **b**) and smartphone photos (**c**, **d**) of the multiplex LFA tested in increasing concentrations (1–1000 ng/mL) of *B. atrox* (**a**, **c**) or *L. muta* (**b**, **d**) venom spiked into plasma (citrate, heparin, K2EDTA), and urine matrices. Calibration curves are plotted as test lines divided by control lines (T/C) ratios (*Bothrops*/control = B/C; *Lachesis*/control = L/C) against concentration. The B/C is not shown for the *L. muta* tests, and the L/C is not shown for the *B. atrox* tests, as no signals developed. Error bars represent the standard deviation (n = 3), and the red cross represents the signal in a blank running buffer.

The LFA was tested in urine and plasma (citrate, heparin, and K2EDTA) matrices spiked with either *B. atrox* (Fig. 3a, c) or *L. muta* (Fig. 3b, d) venom. In all tested matrices, the test line intensities (and corresponding B/C and L/C ratios) decreased with decreasing venom concentration, resulting in a relative standard deviation (%RSD) of < 14.5% at concentrations of 100 ng/mL (Table 2).

**Table 2:** Analytical performance of the multiplex LFA in six biological matrices.

Parameter		Matrix						
		Running buffer	Plasma citrate	Plasma heparin	Plasma K2EDTA	Urine	Serum (clotted blood)	Serum (heat- inactivated)
Dynamic working range (ng/mL)		10–1000	10–1000	10–1000	50–10,000	10–10,000	100–100,000	500–100,000
Sample µL: running buffer µL		10:90	10:90	10:90	10:90	10:90	1:99	1:99
Visual LoD (ng/mL)	Venom concentration before dilution in running buffer	B: 10 L: 50	B: 10 L: 50	B: 10 L: 50	B: 50 L: 50	B: 10 L: 10	B: 100 L: 50	B: 500 L: 500
	Venom concentration after dilution in running buffer	B: 1 L: 5	B: 1 L: 5	B: 1 L: 5	B: 5 L: 5	B: 1 L: 1	B: 1 L: 0.5	B: 5 L: 5
Reproducibility^a^ (n = 3)		B: 11.7% L: 9.61%	B: 14.4% L: 11.0%	B: 8.1% L: 3.1%	B: 1.2% L: 8.3%	B: 7.8% L:11.4%	B: 16.6% L: 14.7%	B: 87.0% L: 39.7%
False positives (n = 20)		0	0	0	2	0	0	0

^a^Reproducibility was calculated as %RSD = *standard deviation/average* × *100* at 100 ng/mL for all matrices, except for the two serum matrices where it was calculated at 500 ng/mL, because this was the lowest concentration detectable in serum (heat-inactivated). B is short for *B. atrox,* and L is short for *L. muta.*

Overall, the test lines were consistently more intense on the *Bothrops* line compared with the *Lachesis* line, resulting in higher B/C than L/C values (Fig. 3). Plasma samples collected in tubes containing different anticoagulants were assessed as it is understood that sample collection can impact assay sensitivity^26^. In the plasma matrices, the *B. atrox* venom resulted in visual LoDs of 10 ng/mL (citrate and heparin) or 50 ng/mL (K2EDTA), while *L. muta* venom consistently gave a LoD of 50 ng/mL. Except for a very weak signal on the *Lachesis* line in plasma K2EDTA (0–10 ng/mL) no other false positives were observed. These results could indicate that plasma K2EDTA is responsible for non-specific binding but could equally be related to variabilities from the individual donor samples. It has been reported that venom concentrations in human samples range from < 1–1000 ng/mL in the first 30 h post-viper bite, and from 10 to 100 ng/mL within the first 10 h^30^. Based on these concentrations, the LFA would be most helpful in the first 10 h following envenoming.

In comparison, LoDs of 10 ng/mL were reached for *B. atrox* and *L. muta* spiked urine, with no false positives in blank urine. The overall signal intensity in spiked urine was comparable to the signal intensity in spiked running buffer. Still, this study used pooled urine, and it is understood that pooled samples have fewer risks in terms of matrix effects^26^. It is expected that urine from individual donors would introduce greater uncertainty. The usefulness of urine for real-time monitoring of venom concentrations is unclear but urine is promising as a non-invasive matrix for reaching the ‘*ease of sample collection*’ aim of the (RE)ASSURED criteria.

### Troubleshooting matrix effects in spiked serum

The LFA was tested in three different dilutions of blank sera (clotted blood and heat-inactivated) in running buffer (diluted 10×, 20×, and 100×), as it is understood that matrix effects can be reduced by diluting the matrix in buffer^47^. In both blank serum matrices, the 10× dilution resulted in the appearance of both test lines. In contrast, the 20× dilution only caused signal development on the *Lachesis* lines, demonstrating that a larger dilution in the running buffer reduced the sera matrix effects. Testing the 100× diluted sera gave true negative results, indicating that a 1:99 µL dilution in running buffer could eliminate the undesirable matrix effects in sera (SI Fig. S7).

**Figure 4:**
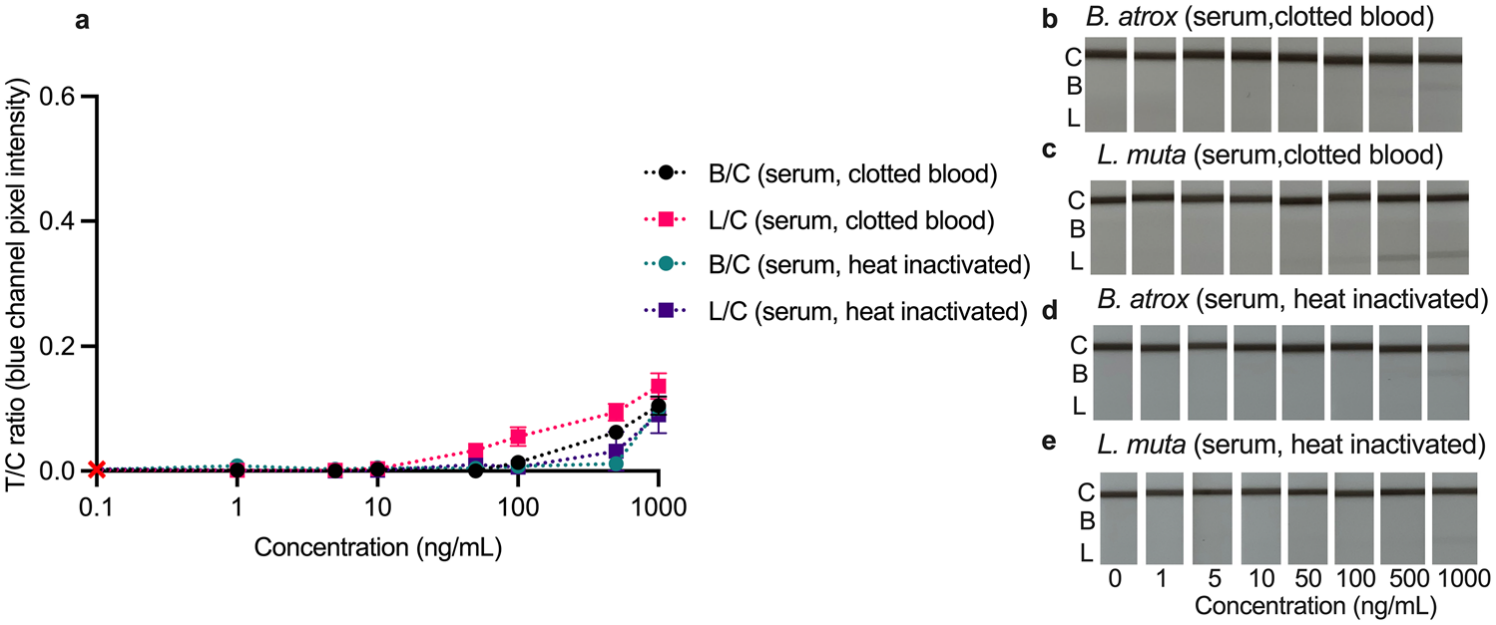
Calibration curves (**a**) and smartphone photos (**b**–**e**) of the multiplex LFA tested in increasing concentrations (1–1000 ng/mL) of *B. atrox* (**b**, **d**) and *L. muta* (**c**, **e**) spiked serum (1 µL spiked serum: 99 µL running buffer). Calibration curves are plotted as test lines divided by control lines (T/C) ratios (*Bothrops*/control = B/C; *Lachesis*/control = L/C) against concentration. The B/C is not shown for the *L. muta* tests, and the L/C is not shown for the *B. atrox* tests, as no signals developed. Error bars represent the standard deviation (n = 3), and the red cross represents the signal in blank running buffer.

The 1:99 ratio eliminated the false positive signals (Fig. 4), but also led to much weaker test line signal intensities, resulting in lower T/C values, and higher %RSDs than other matrices that used a 10:90 ratio (Table 2). Still, these were foreseeable consequences as the venom concentration in the serum was also diluted 100x, reducing the amount of antigen available to bind at the test line. The LFAs tested in serum from clotted blood (Fig. 4a–c) had visual LoDs of 100 ng/mL and 50 ng/mL for *B. atrox* and *L. muta* venom, respectively. In contrast, the visual LoD in the heat-inactivated serum (Fig. 4a, d, e), was 500 ng/mL for both venoms. These LoDs are still within the clinically relevant concentration range following viper bite (< 1–1000 ng/mL)^30^ but would fall short of detecting venoms at the lower end of this range. Based on the more pronounced matrix effects and higher LoDs in sera, it seems plasma or urine would be the preferred matrices for the current LFA. Still, as mentioned, a higher sample dilution was required to eliminate the matrix effects in sera; the venom-spiked sera needed to be diluted by a factor of 100 with running buffer, resulting in an effective lower venom concentration compared with the experiments in other matrices. Therefore, the actual concentrations of venom detected in the experiments with serum were 100× lower than the LoDs reported here (Table 2).

### Evaluation of matrix effects and non-specific binding

**Figure 5:**
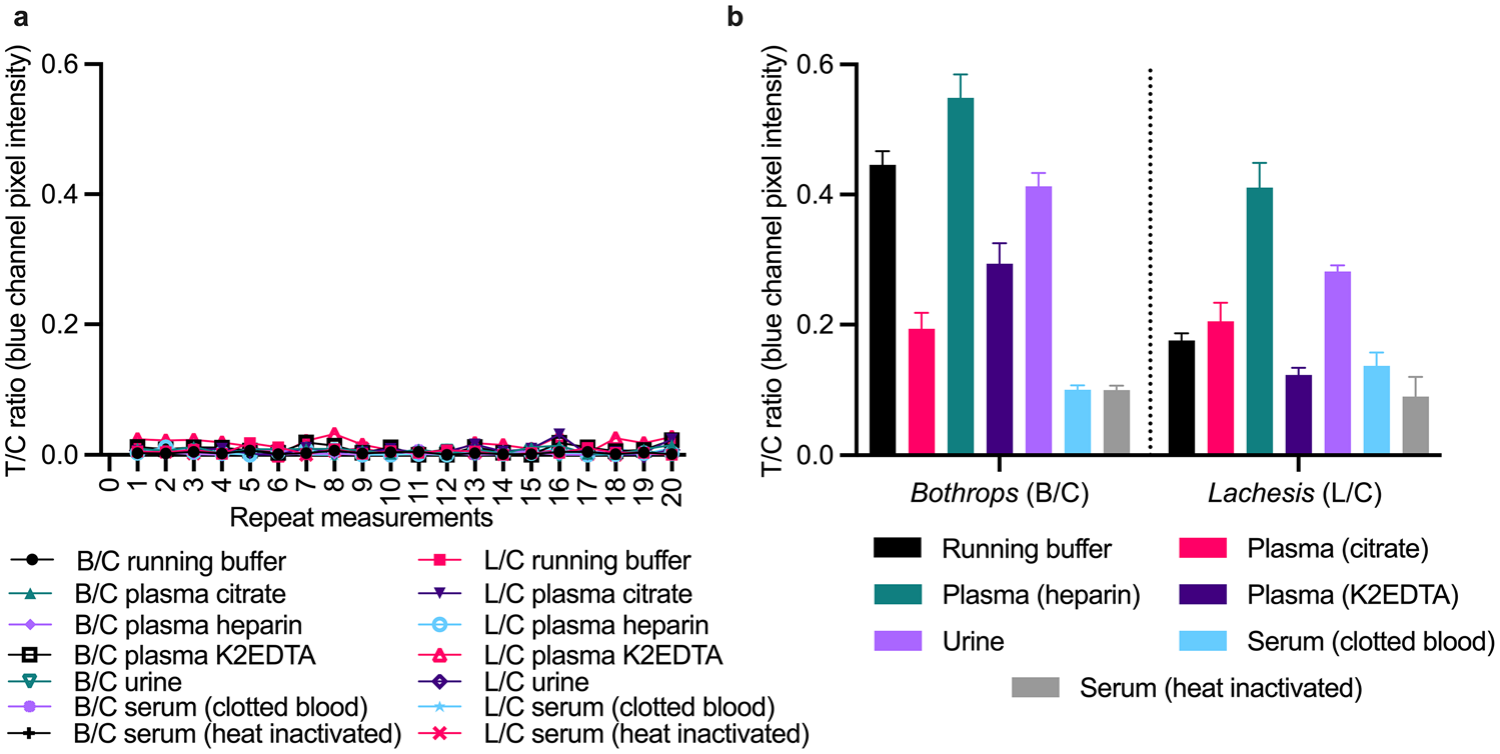
Matrix effects in (**a**) blank sample matrices (n = 20/matrix) and (**b**) at 1000 ng/mL of *B. atrox* or *L. muta* venom spiked into the matrices, plotted as the test line divided by control line (T/C) ratios (*Bothrops*/control = B/C; *Lachesis*/control = L/C) against repeat measurements (**a**), or matrices (**b**). Error bars (**b**) represent the standard deviation (n = 3).

Antibodies are important for assay selectivity but are not infallible and can be subject to non-specific interactions^26^. To understand the impact of non-specific binding on assay performance it is important to evaluate assays in blank matrices. The LFA was tested across repeat (n = 20) blank measurements in running buffer and the six individual biological matrices (n = 140) to evaluate the influence of sample matrix on the assay (Fig. 5a). Matrix effects can influence the analytical performance of an assay in two ways: (1) by increasing non-specific binding leading to an increase in signal intensity in a negative sample, and (2) by influencing the specific binding thereby altering the signal intensity in a positive sample. Only two LFAs out of the 140 tested in blank matrices gave a weak signal on the *Lachesis* line; both these false positives occurred in plasma K2EDTA. While the test line signals in these blanks were extremely weak, further assay optimization would be needed to eliminate them.

No other blank sample matrix caused the appearance of test line signals, demonstrating that the current running buffer composition and sample-to-running buffer ratios (10:90 and 1:99, respectively) were appropriate for eliminating the occurrence of false positives in the tested matrices.

To further understand the influence of the sample matrix on the LFA, B/C and L/C ratios (n = 3) were plotted for each sample matrix spiked with 1000 ng/mL of venom (Fig. 5b). Both the B/C and L/C ratios were higher in spiked plasma heparin compared with spiked running buffer, suggesting that the sample matrix influences the signal development. Likewise, the L/C was higher in plasma citrate and urine compared with the running buffer. In comparison, these same matrices yielded a lower B/C, demonstrating that matrix type influenced the test lines’ development differently. This influence could be due to interference from the anticoagulant used in the tube for storing the sample or because the samples are from individual donors. More samples from individual donors should be tested to better understand the extent of the matrix effects on the LFA. Parameters related to the analytical performance of the LFA are summarized in Table 2.

### Assessing the influence of high antigen concentration

**Figure 6:**
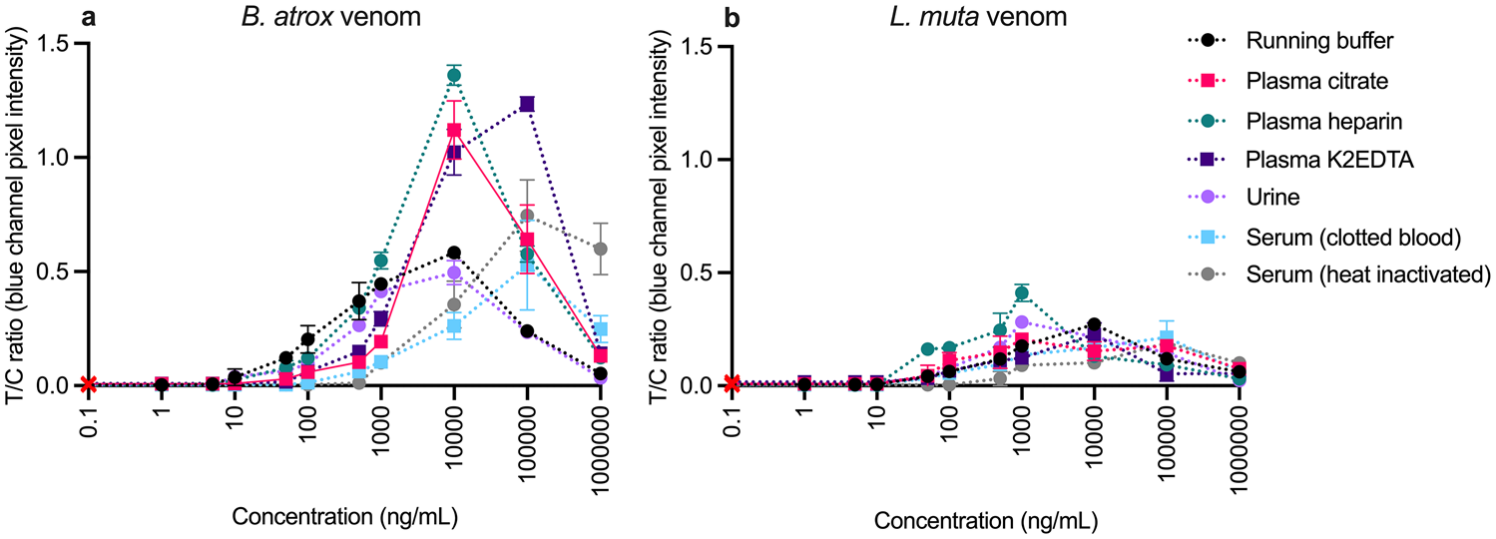
Multiplex LFA tested in an extended high antigen concentration range (1–1,000,000 ng/mL) in all sample matrices. Calibration curves plotted as test line divided by control line (T/C) ratios (*Bothrops*/control = B/C; *Lachesis*/control = L/C) against *B. atrox* (**a**) or *L. muta*, (**b**) venom concentrations. Error bars represent the standard deviation (n = 3 for 0–1000 ng/mL; n = 2 for 10,000–1,000,000 ng/mL), and the red cross represents the signal in a blank.

Within a sandwich LFA’s dynamic working range, the test line intensity increases with increasing antigen concentrations, corresponding to an increasing T/C ratio (Fig. 6). However, at concentrations above this range, the test line intensity (and T/C ratio) starts to decrease, leading to falsely low results. At excess concentrations, the hook effect can occur, where both the test line antibodies and the detection antibodies can become saturated with antigen, preventing binding of the detection antibody at the test line^40^. These effects occur at different concentrations depending on the specific assay and conditions tested.

The *Bothrops* line signal intensity and corresponding B/C ratio decreased at concentrations above 10,000 ng/mL in running buffer, plasma (citrate, heparin), and urine, or above 100,000 ng/mL in plasma (K2EDTA) and both sera (Fig. 6a). In contrast, the *Lachesis* line’s intensity decreased at concentrations above 1000 ng/mL in plasma (citrate, heparin) and urine, above 10,000 ng/mL in running buffer and plasma K2EDTA, and above 100,000 ng/mL in both sera (Fig. 6b). It is logical that the loss of test line occurred at higher antigen concentrations in sera as these were 10× more diluted than the other matrices. In all matrices, positive test line signals were still visible at 1,000,000 ng/mL, although the intensities of these signals were weak and indistinguishable from those at much lower concentrations (SI Fig. S8). In addition to causing a reduction in signal intensity, the high antigen concentrations also caused a delay in the signal development time, with signals at the highest concentrations only starting to develop after 10 min.

Based on the literature it is unlikely that the venom concentrations in bodily fluids would be high enough to cause the hook effect^30^ in the developed assay. However, it has been reported that venom concentrations are higher in wound exudate or blister samples^48^. It is possible that venom concentrations at a bite site could be high enough to cause the hook effect. Therefore, considerable testing with wound swabs would be required before including this as a potential sample matrix.

Importantly, no signal developed on the *Bothrops* line in an excess of *L. muta* venom, or vice versa in any of the tested matrices, even at these extreme concentrations, indicating the usefulness of the LFA for differentiating between these venoms across a wide concentration range.

### Smartphone-based biosensor potential

Smartphones, combined with mechanisms to control lighting conditions for standardizing image capture, have emerged as a popular tool for acquiring and processing raw data from optical measurements, such as signal intensities from LFAs that can be correlated with antigen concentrations^24^. The LFA results were more readable by the naked eye than by smartphone, but visual readout is subjective and untrained users may misinterpret ambiguous results. Here, the smartphone was used solely to record data to be analyzed in ImageJ. However, a future improvement could be implementing on-smartphone processing, for real-time result interpretation and transmission to relevant healthcare personnel or storage in medical records, thereby, working towards the (RE)ASSURED criteria.

## Conclusion

Efficient management is essential to limit the devastating effects of venom on snakebite victims, yet current methods for diagnosing snakebites rely on the knowledge of highly specialized medical personnel. Fast and affordable analytical tools that accurately identify and differentiate venoms in bodily fluids could make venom detection more accessible in hospital PoC settings and rural healthcare clinics. Moreover, in the future such a tool could potentially be useful to dose antivenom. If the LFA still detects venom in a patient’s biological fluids hours after antivenom administration, it might indicate the need for more vials of antivenom. Still, the LFA would require extensive testing with samples containing antivenom before determining such a use case. In this study, an LFA prototype capable of detecting *Bothrops* and *Lachesis* spp. venom components in various bodily fluids was developed and the necessary dilution factors to prevent matrix effects were determined. The assay LoDs are clinically relevant for average venom concentrations, but further optimization is required to ensure detection at the lower end of this range. As the LFA was developed using mAbs targeting single venom toxins rather than whole venoms, the actual concentrations of the detected toxins are lower than the whole venom concentrations reported here. Individual differences in donor samples also influence the results. Therefore, further validation must be carried out with spiked matrices from numerous individual donors and, crucially, real-life samples from envenomed patients. The current prototype is based on a half-dipstick format. To make it more user-friendly, future iterations of the LFA would include a conjugate pad with the CNP-mAbs dried down as well as a sample pad so that the technician, nurse, or other hospital personnel using the test only needs to apply the sample diluted in running buffer directly to the sample pad. Moreover, the usability could be further improved by including a disposable dropper bottle or single-use pipette for applying the diluted sample to the test so that laboratory pipettes are not required. Additionally, conducting user experience studies with the intended end-users in a real-life setting is vital to determine how well the technology could be adopted. With further optimization and validation, this LFA could develop into a valuable diagnostic tool to support clinical snakebite management in Brazil.

## Materials and methods

### Reagents and consumables

The washing buffer was composed of 5 mM borate buffer (pH 8.8) diluted from a mixture of 100 mM sodium tetraborate, 100 mM boric acid (Sigma Aldrich, Switzerland), and IgG and protease-free bovine serum albumin (BSA; Jackson Immunoresearch, Denmark) added to a final concentration of 1% (*w*/*v*) as a blocking agent. Storage buffer consisted of 100 mM borate buffer with 1% BSA (*w*/*v*) as a blocking agent. Running buffers were prepared by adding 1% BSA (*w/v*) as a blocking agent and either (1) 0.05% Tween-20 (*v/v*) (ThermoFischer, Denmark), or (2) 0.5% NP-40 Tergitol (*v/v*) (Sigma Aldrich, Switzerland), with or without the addition of 4% NaCl (*w/v*) (ThermoFischer, Denmark). All solutions were prepared with water from a MilliQ-system (MQ) (> 18.2 MΩ/cm) from Millipore (Burlington, MA, USA).

### Venoms

Lyophilized whole venoms were purchased from Latoxan (France) or donated by scientists SI Table S2). Whole venoms were weighed (2 mg) using an analytical balance (Sartorius, Germany) and subsequently dissolved in 1 mL of PBS (pH 7.4; 0.14 M). The resulting venom concentrations were calculated based on the weighed mass of the lyophilized venoms in the measured buffer volume.

### Matrices

The matrices used in this study (plasma (citrate, heparin, and K2EDTA), sera (heat-inactivated and from whole clotted blood), and urine) are summarized in SI Table S3. All matrices were aliquoted, stored at − 20 °C, and defrosted on the day of the experiment. The matrices used in the study were free (blank) from the target antigens. The blank matrices were spiked with whole venoms at a starting concentration of 1 mg/mL, and then a 10-fold dilution into the matrix was carried out until a concentration of 1 ng/mL. The spiked matrices in the concentration range of 1000, 100, 10, 1 ng/mL were further diluted (10:90, or 1:99) into the running buffer before analysis.

### Antibody discovery and production

The *Lachesis*-mAb sandwich pair were discovered using the method previously reported for the *Bothrops-*mAb pair^38^. All protocols in this study were approved by the Research Ethical Committee of University of Southern Denmark, following the guidelines of the Danish National Animal Ethics Committee (J-Number 2015-15-0201-00680), and were performed according to the ARRIVE guidelines. A special effort was made to keep the necessary number of animals to a minimum. In brief, mice were immunized with *L. muta* whole venom and the generated hybridoma cell lines were cloned to monoclonality. The affinity-purified mAbs were tested for binding to 21 whole venoms (SI Table S2) in indirect ELISAs. Antibodies that only reacted strongly with venom from *Lachesis* spp. were tested against 121 mAbs raised against the venoms of either *B. atrox*, *L. muta*, or *C. d. terrificus* in sandwich ELISAs to identify sandwich pairs. All identified sandwich pairs were screened in indirect sandwich ELISAs to assess their ability to specifically detect 21 different venoms.

### Coupling antibodies to carbon nanoparticles

The carbon coupling was carried out as previously reported^39^. Antibodies were buffer exchanged from PBS (pH 7.4) into 5 mM borate buffer (pH 8.8) using Zeba Spin Protein Desalting columns (ThermoScientific, Denmark). The antibody concentrations were measured using a NanoDrop (ThermoScientific, Denmark) before being coupled with ‘Orion Special Black 4’ carbon nanoparticles (CNPs) kindly provided by Grolman Nordic Specialty Chemicals (Lystrup, Denmark). A 1% suspension of CNPs was prepared by adding 1 mL of MQ Water to 10 mg carbon and sonicating for 10 min. The 1% suspension was diluted 5× in 5 mM borate buffer (pH 8.8) to obtain a 0.2% suspension, which was sonicated for 5 min. Next, 350 µg of purified 86–11 (*Bothrops*) or 83–17 (*Lachesis*) antibody solution was added to 1 mL of 0.2% carbon suspension and stirred overnight at 4 °C. The suspension was split into equal aliquots, and 500 µL of washing buffer was added to each before centrifuging for 15 min at 13,636 × *g* at 4 °C. The supernatants were discarded, and the pellets were re-suspended in washing buffer. This process was repeated twice. Finally, the supernatants were discarded, and the coupling reaction was finished by pooling the CNP-mAb pellets with 1 mL storage buffer containing 1% BSA as a blocking agent and stored at 4 °C until use.

### Singleplex and multiplex LFA development

The control line antibody (goat anti-mouse IgG in PBS (pH 7.6) (1.3 mg/mL; AffiniPure F(ab’)_2_ Fragment specific) (Jackson Immunoresearch Laboratories Inc, Denmark)) and buffer exchanged test line antibodies were diluted in 5 mM borate buffer with 1% D(+)-trehalose dihydrate (*w/v*) (Carl Roth GmbH, Germany) before being dispensed onto backed nitrocellulose HF135NC membrane cards (flow rate of 135 s/4 cm; Merck, Germany), overlaid with an absorbent pad (Whatman, GE Healthcare, Darmstadt, Germany). All LFAs were prepared using a XYLite BioDot dispensing platform (Irvine, California, USA) using dispensing conditions of 1.5 µL/cm and a dispensing rate of 20 mm/s. The singleplex LFAs were prepared by dispensing 0.5 mg/mL of control antibody 10 mm from the absorbent pad and 0.5 mg/mL of either *Bothrops* mAb (86-14) or *Lachesis* mAb (83-02) 5 mm from the control line. Using the same antibody concentrations and spraying conditions, two multiplex LFA configurations were prepared with each line 5 mm apart, one with the configuration control line, *Bothrops* line, *Lachesis* line, (configuration 1), and the other with control line, *Lachesis* line, and *Bothrops* line (configuration 2). After dispensing, LFAs were dried at 37 °C for 1 h and were cut to 4 mm using the BioDot guillotine (Irvine California, USA). LFAs were stored with silica desiccant pouches in heat-sealed foil bags until use. The membranes were not blocked during prototype development but blocking agents were incorporated in the assay running buffer to enable blocking of the membrane while running the assay. See SI Table S4 for an overview of experiments performed with the developed LFAs.

### Optimization and selection of LFA running buffer

The *Bothrops* LFAs were tested in four different blank matrices (*i.e.*, serum from whole clotted blood, heat-inactivated serum, plasma citrate, and plasma heparin) using running buffers with different surfactants (*i.e.*, Tween-20 or Tergitol), with or without the presence of 4% NaCl. The *Lachesis* LFAs were tested under the same conditions but only in running buffers containing 4% NaCl. The LFAs (n = 2) were inserted into microwells containing 1 µL of CNP-mAb, 90 µL of running buffer, and 10 µL of blank matrix.

### Determination of the visual limit of detection for singleplex LFAs

The singleplex *Bothrops* and *Lachesis* LFAs were tested in decreasing concentrations (1000, 500, 100, 50, 10, 5, 1, and 0 ng/mL) of *B. atrox* or *L. muta* venom-spiked running buffer. Both sets of LFAs (n = 3) were inserted into microwells containing 1 µL of either CNP-*Bothrops* or CNP-*Lachesis,* 90 µL of running buffer, and 10 µL venom-spiked running buffer.

### Comparison of multiplex LFA configurations in spiked running buffer

To decide the optimal test line configuration, both LFA configurations were first tested in logarithmically decreasing concentrations (1000, 100, 10, 1, and 0 ng/mL) of (1) both venoms, (2) *B. atrox* venom, or (3) *L. muta* venom spiked into running buffer. The optimal configuration was then further tested in a larger concentration range (1000, 500, 100, 50, 10, 5, 1, and 0 ng/mL) of the venom-spiked running buffer. The LFAs (n = 3) were inserted into microwells containing 0.5 µL of CNP-*Bothrops*-mAb, 0.5 µL CNP-*Lachesis*-mAb, 90 µL running buffer, and 10 µL venom-spiked running buffer.

### Cross-reactivity testing with the optimal multiplex LFA configuration

The optimal LFA configuration was tested with 13 different venoms (SI Table S2) spiked into running buffer at concentrations of 100 and 1000 ng/mL. The LFAs (n = 2) were inserted into microwells containing 0.5 µL CNP-*Bothrops*, 0.5 µL CNP-*Lachesis*, 90 µL of running buffer, and 10 µL spiked running buffer.

### Testing optimal multiplex LFA in spiked urine & plasma

The LFA was tested in decreasing concentrations (1000, 500, 100, 50, 10, 5, 1, and 0 ng/mL) of (1) *B. atrox* venom or (2) *L. muta* venom spiked into urine or different plasma (citrate, heparin, or K2EDTA) matrices. LFAs (n = 3) were inserted into microwells containing 0.5 µL CNP-*Bothrops*, 0.5 µL CNP-*Lachesis*, 90 µL running buffer, and 10 µL spiked urine or plasma.

### Troubleshooting and testing optimal multiplex LFA in spiked serum

The LFA was tested in two different blank serum matrices (*i.e.*, heat-inactivated and from clotted blood) using different sample-to-running buffer ratios. The LFAs (n = 2) were inserted into microwells containing 0.5 µL CNP-*Bothrops*, 0.5 µL CNP-*Lachesis* and either (1) 1 µL of serum:99 µL of running buffer, (2) 5 µL of serum:95 µL of running buffer, or (3) 10 µL of serum:90 µL of running buffer. Using the 1:99 ratio, the LFA was then tested (n = 3) in decreasing concentrations (1000, 500, 100, 50, 10, 5, 1, and 0 ng/mL) of (1) *B. atrox* venom or (2) *L. muta* venom spiked in the different sera.

### Matrix effects

The LFA was tested in each matrix across 20 repeat blank measurements. The LFAs (n = 20/per matrix) were inserted into individual microwells containing 0.5 µL of CNP-*Bothrops,* 0.5 µL CNP-*Lachesis,* and either (1) 10 µL of blank running buffer or blank matrix (plasma (citrate, heparin, K2EDTA), or urine) in 90 µL running buffer or (2) 1 µL of blank sera (clotted blood or heat-inactivated) in 99 µL of running buffer.

### High antigen concentration in spiked matrix experiments

The LFA was tested at excess venom concentrations in each matrix. The LFAs (n = 2) were inserted into individual microwells containing 0.5 µL of CNP-*Bothrops,* 0.5 µL CNP-*Lachesis* and either (1) 10 µL of venom-spiked running buffer or matrix (plasma (citrate, heparin, K2EDTA), or urine) and 90 µL of running buffer or (2) 1 µL of venom-spiked matrix sera (clotted blood or heat-inactivated) in 99 µL running buffer at concentrations of 10,000, 100,000, or 1,000,000 ng/mL.

### LFA readout

In all experiments, the LFAs were left to develop for 15 min, after which the strips were removed, and read by naked eye (qualitative assessment) and photographed by a smartphone camera under controlled lighting conditions (semi-quantitative assessment). Free computer-aided design (CAD) software Autodesk Fusion 360 (Autodesk Inc, California, USA) was used for designing a custom smartphone lightbox and insert for holding up to seven (4 mm wide) LFAs. The designs were saved as .stl files and PrusaSlicer 2.5.2 was used to convert the files to a printable format (Gcode). A fused deposition modeling (FDM) model Prusa Mini + (Prusa Research, Czech Republic) printed the parts in PLA filament (Internet of Printing, The Netherlands) at 0.2 mm resolution. See SI Fig. S9 for an overview of the 3D-printed platform. A smartphone (Huawei P20, Huawei Technologies, China) using the OpenCamera (v1.47.30) app was attached to the lightbox and used to record photos of the developed LFAs. The photos were split into RGB (red, green, blue) color channels using ImageJ^49^. Blue channel pixel intensity readings were recorded from the LFA background, control line, and test line(s); control and test line readings were subtracted from the background to give corrected blue channel pixel intensities. The test line signal intensities were then divided by their corresponding control lines to give T/C ratios, referred to as B/C (*Bothrops*/control) or L/C (*Lachesis*/control). See SI Protocol S1 for an overview of the ImageJ method used. Visual LoDs were established as the minimum antigen concentration that produced a test line signal that could be distinguished from the signal in a blank.

### Supplementary Information

Supplementary Information.

## Data Availability

The datasets generated during and/or analysed during the current study are available from the corresponding author upon reasonable request.

## References

[1] Secretaria De Vigilância em Saúde, Ministério da Saúde. Boletim Epidemiológico: Vigilância epidemiológica do sarampo no Brasil 2020: Semana Epidemiológica. https://www.gov.br/saude/pt-br/assuntos/saude-de-a-a-z/a/animais-peconhentos/acidentes-ofidicos (2020). Accessed 5 Nov 2023.

[2] Schneider MC, Min KD, Hamrick PN (2021). Overview of snakebite in Brazil: Possible drivers and a tool for risk mapping. PLoS Negl. Trop. Dis..

[3] Bochner, R., Fiszon, J. T. & Machado, C. A profile of snake bites in Brazil 2001 to 2012. *J. Clin. Toxicol.* **4**. 10.4172/2161-0495.1000194 (2014).

[4] da Silva WRGB, de Siqueira SL, Lira D (2023). Who are the most affected by *Bothrops* snakebite envenoming in Brazil? A Clinical-epidemiological profile study among the regions of the country. PLoS Negl. Trop. Dis..

[5] Knudsen C, Jürgensen JA, Føns S (2021). Snakebite envenoming diagnosis and diagnostics. Front. Immunol..

[6] Strand E, Murta F, Tupetz A (2023). Perspectives on snakebite envenoming care needs across different sociocultural contexts and health systems: A comparative qualitative analysis among US and Brazilian health providers. Toxicon X.

[7] Mise YF, Lira-da-Silva R, Carvalho FM (2018). Time to treatment and severity of snake envenoming in Brazil. Rev. Panam. Salud Públ..

[8] Feitosa EL, Sampaio VS, Salinas JL (2015). Older age and time to medical assistance are associated with severity and mortality of snakebites in the Brazilian Amazon: A case-control study. PLoS One.

[9] Feitosa ES, Sampaio V, Sachett J (2015). Snakebites as a largely neglected problem in the Brazilian Amazon: Highlights of the epidemiological trends in the state of Amazonas. Rev. Soc. Bras. Med. Trop..

[10] Magalhães SFV, Peixoto HM, Moura N (2019). Snakebite envenomation in the Brazilian Amazon: A descriptive study. Trans. R. Soc. Trop. Med. Hyg..

[11] Puzari U, Mukherjee AK (2020). Recent developments in diagnostic tools and bioanalytical methods for analysis of snake venom: A critical review. Anal. Chim. Acta.

[12] Liu CC, Yu JS, Wang PJ (2018). Development of sandwich ELISA and lateral flow strip assays for diagnosing clinically significant snakebite in Taiwan. PLoS Negl. Trop. Dis..

[13] Anand A, Chatterjee B, Dhiman A (2021). Complex target SELEX-based identification of DNA aptamers against *Bungarus caeruleus* venom for the detection of envenomation using a paper-based device. Biosens. Bioelectron..

[14] Chinonavanig L, Karnchanachetanee C, Pongsettakul P, Ratanabanangkoon K (1991). Diagnosis of snake venoms by a reverse latex agglutination test. J. Clin. Toxicol..

[15] Van Dong L, Khoo HE, Quyen LK, Gopalakrishnakone P (2004). Optical immunoassay for snake venom detection. Biosens. Bioelectron..

[16] Chen F, Qin M, Liu W (2021). Snake venom identification via fluorescent discrimination. Anal. Chem..

[17] Mitra I, Roy S, Haque I (2022). Forensic identification of four Indian snake species using single multiplex polymerase chain reaction. J. Forens. Sci. Med..

[18] Agurto-Arteaga A, Vivas-Ruiz DE, Lazo F (2023). Simultaneous identification of three clinically relevant peruvian pit vipers by multiplex loop-mediated isothermal amplification (mLAMP). Toxicon.

[19] Dorledo de Faria RA (2020). Label-free impedimetric immunosensors for detection of snake venoms using polyaniline as a transducer substrate. Biomed. J. Sci. Tech. Res..

[20] Macêdo JKA, Joseph JK, Menon J (2019). Proteomic analysis of human blister fluids following envenomation by three snake species in India: Differential markers for venom mechanisms of action. Toxins.

[21] Nimorakiotakis V, Winkel KD (2016). Prospective assessment of the false positive rate of the Australian snake venom detection kit in healthy human samples. Toxicon.

[22] World Health Organization. Control of neglected tropical diseases: Snakebite envenoming treatment. https://www.who.int/teams/control-of-neglected-tropical-diseases/snakebite-envenoming/treatment (2023). Accessed 18 Oct 2023.

[23] World Health Organization. Mapping the landscape of diagnostics for sexually transmitted infections: Key findings and recommendations. https://iris.who.int/handle/10665/68990?locale-attribute=en& (2004). Accessed 6 Sept 2023.

[24] Ross, G. M. S. *et al.* Best practices and current implementation of emerging smartphone-based (bio)sensors—part 1: Data handling and ethics. *TrAC Trends Anal. Chem.* **158**. 10.1016/j.trac.2022.116863 (2023).

[25] Otoo JA, Schlappi TS (2022). REASSURED multiplex diagnostics: A critical review and forecast. Biosens.

[26] Masson JF (2020). Consideration of sample matrix effects and “biological” noise in optimizing the limit of detection of biosensors. ACS Sens..

[27] Williams HF, Layfield HJ, Vallance T (2019). The urgent need to develop novel strategies for the diagnosis and treatment of snakebites. Toxins.

[28] Williams HF, Vaiyapuri R, Gajjeraman P (2017). Challenges in diagnosing and treating snakebites in a rural population of Tamil Nadu, India: The views of clinicians. Toxicon.

[29] Brown NI (2012). Consequences of neglect: Analysis of the sub-saharan african snake antivenom market and the global context. PLoS Negl. Trop. Dis..

[30] Sanhajariya S, Duffull SB, Isbister GK (2018). Pharmacokinetics of snake venom. Toxins.

[31] Liu CC, Yang YH, Hsiao YC (2021). Rapid and efficient enrichment of snake venoms from human plasma using a strong cation exchange tip column to improve snakebite diagnosis. Toxins.

[32] Hung D-Z, Lin J-H, Mo JF (2014). Rapid diagnosis of *Naja atra* snakebites. Clin. Toxicol..

[33] Pawade BS, Salvi NC, Shaikh IK (2016). Rapid and selective detection of experimental snake envenomation—use of gold nanoparticle based lateral flow assay. Toxicon.

[34] Lin J-H, Sung W, Liao J, Hung D-Z (2020). Rapid and international applicable diagnostic device for cobra (Genus* Naja*) snakebites. Toxins.

[35] Lin J-H, Lo C-M, Chuang S-H (2020). Collocation of avian and mammal antibodies to develop a rapid and sensitive diagnostic tool for Russell’s vipers snakebite. PLoS Negl. Trop. Dis..

[36] Kaul S, Keerthana LS, Kumar P (2021). Cytotoxin antibody-based colourimetric sensor for field-level differential detection of elapid among big four snake venom. PLoS Negl. Trop. Dis..

[37] Nong JF, Huang Z, Huang ZZ (2023). Development of sandwich ELISA and lateral flow assay for the detection of *Bungarus multicinctus* venom. PLoS Negl. Trop. Dis..

[38] Knudsen C, Jürgensen JA, Knudsen DP (2023). Prototyping of a lateral flow assay based on monoclonal antibodies for detection of *Bothrops* venoms. Anal. Chim. Acta.

[39] Ross GMS, Bremer MGEG, Wichers JH (2018). Rapid antibody selection using surface plasmon resonance for high-speed and sensitive hazelnut lateral flow prototypes. Biosens..

[40] Ross GMS, Filippini D, Nielen MWF, Salentijn GIJ (2020). Unraveling the Hook effect: A comprehensive study of high antigen concentration effects in sandwich lateral flow immunoassays. Anal. Chem..

[41] Ejazi SA, Choudhury ST, Bhattacharyya A (2021). Development and clinical evaluation of serum and urine-based lateral flow tests for diagnosis of human visceral leishmaniasis. Microorganisms.

[42] Güven E, Duus K, Lydolph MC (2014). Non-specific binding in solid phase immunoassays for autoantibodies correlates with inflammation markers. J. Immunol. Methods.

[43] Ward G, Simpson A, Boscato L, Hickman PE (2017). The investigation of interferences in immunoassay. Clin. Biochem..

[44] Madrigal M, Sanz L, Flores-Díaz M (2012). Snake venomics across genus *Lachesis*. Ontogenetic changes in the venom composition of *Lachesis stenophrys* and comparative proteomics of the venoms of adult *Lachesis melanocephala* and *Lachesis acrochorda*. J. Proteom..

[45] Rodrigues CFB, Zdenek CN, Bourke LA (2021). Clinical implications of ontogenetic differences in the coagulotoxic activity of *Bothrops jararacussu* venoms. Toxicol. Lett..

[46] Calvete JJ, Sanz L, Pérez A (2011). Snake population venomics and antivenomics of *Bothrops atrox*: Paedomorphism along its transamazonian dispersal and implications of geographic venom variability on snakebite management. J. Proteom..

[47] Huang L, Zhang F, Li F (2022). Development of Ic-ELISA and colloidal gold lateral flow immunoassay for the determination of cypermethrin in agricultural samples. Biosens..

[48] Van DL, Quyen LK, Khoo HE, Gopalakrishnakone P (2003). Immunogenicity of venoms from four common snakes in the South of Vietnam and development of ELISA kit for venom detection. J. Immunol. Methods.

[49] Schneider CA, Rasband WS, Eliceiri KW (2012). NIH image to ImageJ: 25 years of image analysis. Nat. Methods.

