# Supplementary material for: Multiplex lateral flow assay development for snake venom detection in biological matrices
Source: Sci Rep. 2024 Jan 31;14:2567. doi: 10.1038/s41598-024-51971-2 (PMC10831076; doi:10.1038/s41598-024-51971-2)

Multiplex lateral flow assay development for snake venom detection in biological matrices

Cecilie Knudsen[1,2†*], Selma B. Belfakir[1,2†*], Pelle Degnegaard[1†], Jonas A. Jürgensen[1,2], Aleksander M. Haack[1,2], Rasmus U. W. Friis[1,2], Søren H. Dam[1,2], Andreas H. Laustsen[1,2], Georgina M. S. Ross[1*]

[1] VenomAid Diagnostics, DK-2800 Kongens Lyngby, Denmark
[2] Department of Biotechnology and Biomedicine, Technical University of Denmark, DK-2800 Kongens Lyngby, Denmark

[†] These authors contributed equally to this work and are co-first authors
*Corresponding authors: ckn@venomaid.com; selbel@dtu.dk; gin@venomaid.com

Table of contents

**Fig. S1**	Antibody sandwich pair selection by indirect sandwich ELISA	S2
**Fig. S2**	Photos of singleplex *Bothrops* LFA in blank matrices using running buffers with/without NaCl	S2
**Table S1**	Comparison of *Bothrops* LFA in blank sample matrices with different running buffer compositions	S2
**Fig. S3**	Photos of singleplex *Lachesis* LFA in blank matrices in different running buffers with NaCl	S3
**Fig. S4**	Photos and calibration curves of *Bothrops* and *Lachesis* singleplex LFAs in spiked running buffer	S3
**Fig. S5**	Calibration curves comparing the two multiplex LFA configurations in spiked running buffer	S4
**Fig. S6**	Photos of the two multiplex LFA configurations tested in different blank matrices	S4
**Table S2**	List of snake venoms used in this study	S5
**Fig. S7**	Photos of the optimal multiplex LFA configuration tested in different serum-to-running buffer ratios	S5
**Fig. S8**	Photos and bar chart of multiplex LFA tested at high antigen concentrations	S6
**Table S3**	List of matrix samples used in this study	S6
**Table S4**	Overview of experiments performed with the developed LFAs	S7
**Fig. S9**	3D printed platform for reading LFAs by smartphone	S7
**Protocol S1**	(Semi)quantitatively analyzing LFAs using ImageJ	S8

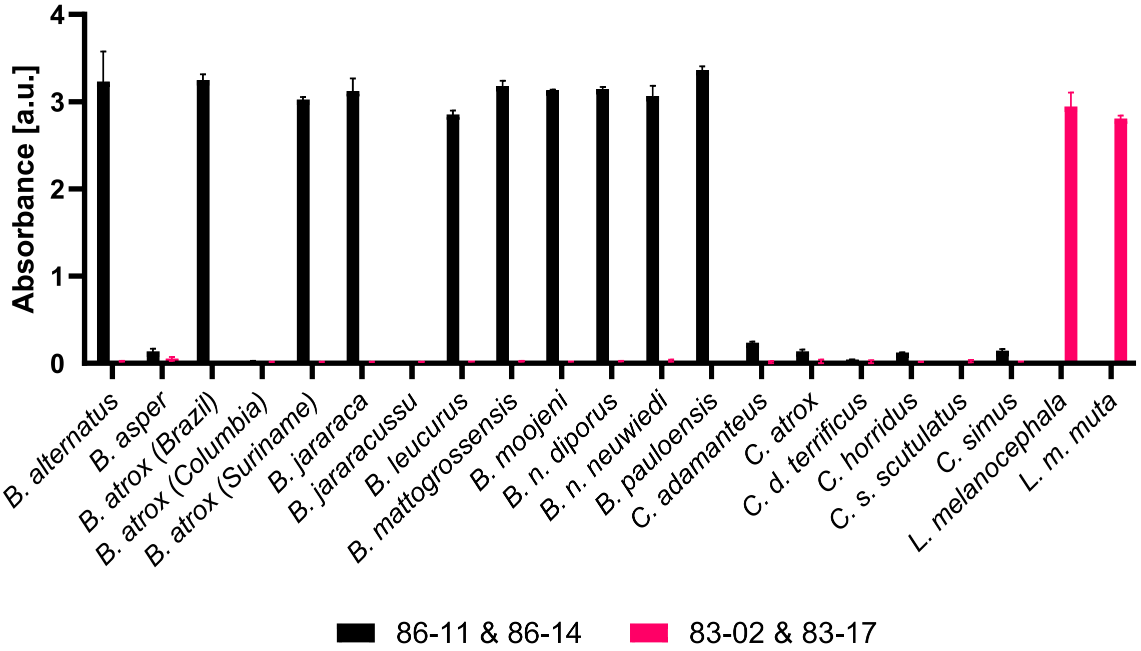

Fig. S1 The ability of the *Bothrops* sandwich pair (consisting of antibodies 86-11 and 86-14) and *Lachesis* sandwich pair (consisting of antibodies 83-02 and 83-17) to bind 21 different venoms at 1000 ng/mL venom was assessed in indirect sandwich ELISAs. Error bars indicate standard deviations from duplicate measurements.

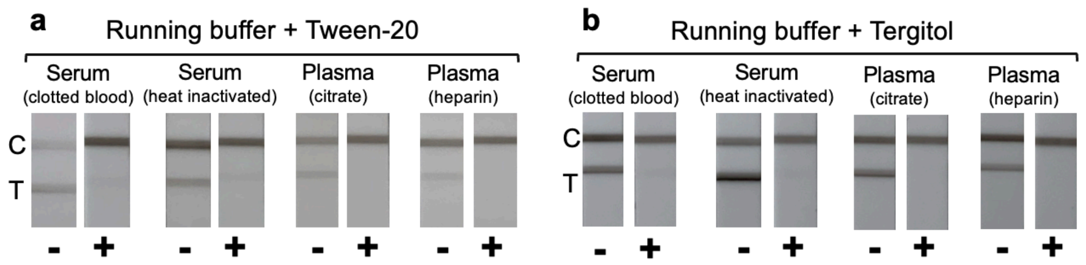

Fig. S2 Photos of the singleplex *Bothrops* LFA tested in four blank matrices using different running buffer compositions without (-) or with (+) the addition of 4% NaCl; (a) LFAs in running buffer with Tween-20; (b) LFAs in running buffer with Tergitol.

Table S1 Comparison of *Bothrops* LFA in blank sample matrices with different running buffer compositions

	Serum (clotted blood)	Serum (heat-inactivated)	Plasma (citrate)	Plasma (heparin)
+ Tween-20	False positive	False positive	False positive	False positive
+ Tween-20 + NaCl	False positive	False positive	True negative	True negative
+ Tergitol	False positive	False positive	False positive	False positive
+ Tergitol + NaCl	False positive	False positive	True negative	True negative

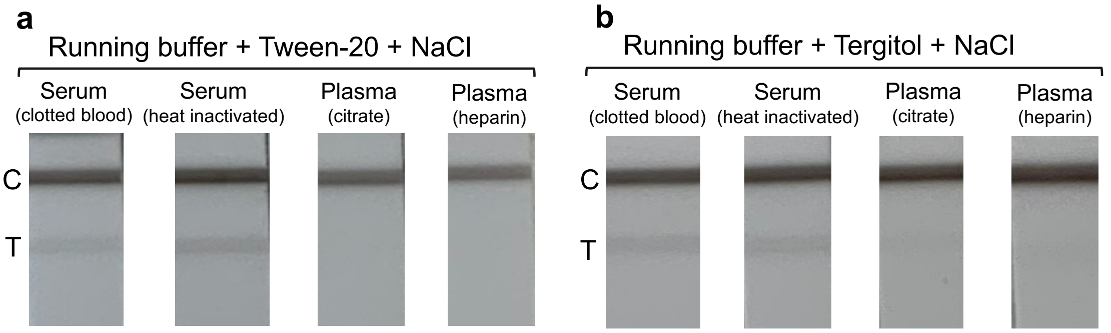

Fig. S3 Photos of the singleplex *Lachesis* LFA tested in four blank matrices using different running buffer combinations with the addition of 4% NaCl; (a) LFAs in running buffer with Tween-20; (b) LFAs in running buffer with Tergitol.

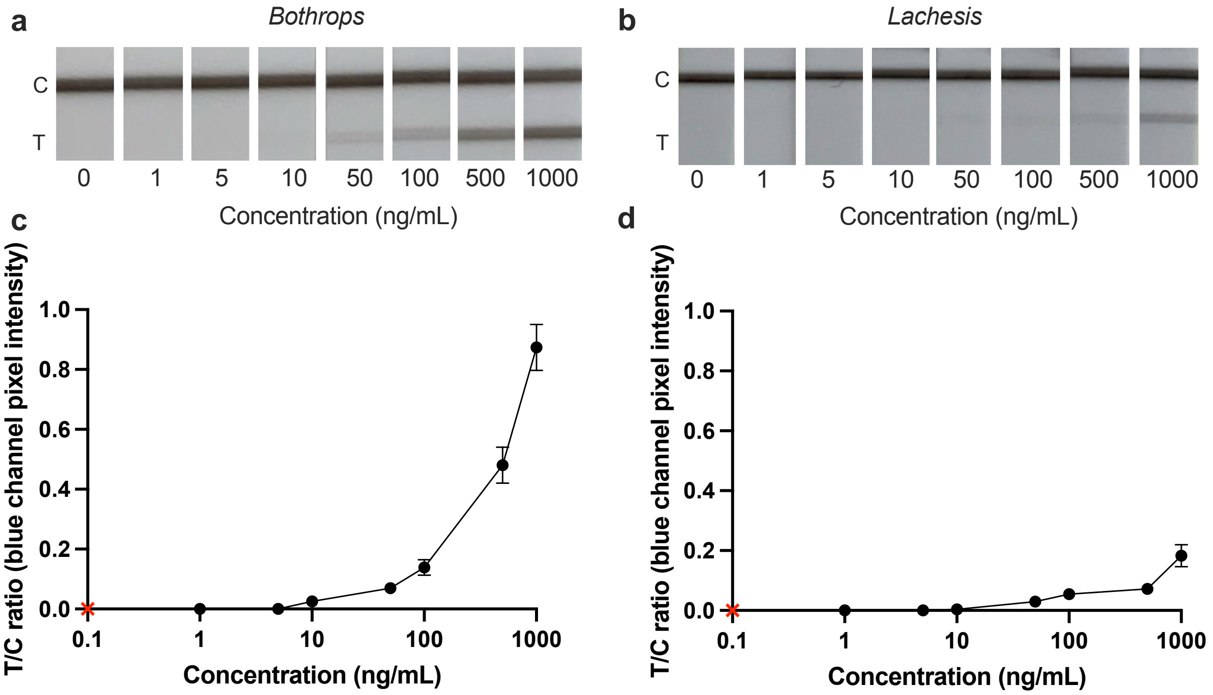

Fig. S4 Photographs and calibration curves showing the *Bothrops* and *Lachesis* singleplex LFAs tested in increasing concentrations (1-1000 ng/mL) of their respective venoms in running buffer; (a) photos of *Bothrops* LFAs in running buffer spiked with *B. atrox* venom; (b) photos of *Lachesis* LFAs in running buffer spiked with *L. muta* venom; (c) calibration curves for *Bothrops* LFAs in spiked running buffer, (d) calibration curves for *Lachesis* LFAs in spiked running buffer. Error bars represent the standard deviation (n=3), and the red cross represents the signal in a blank sample (0 ng/mL).

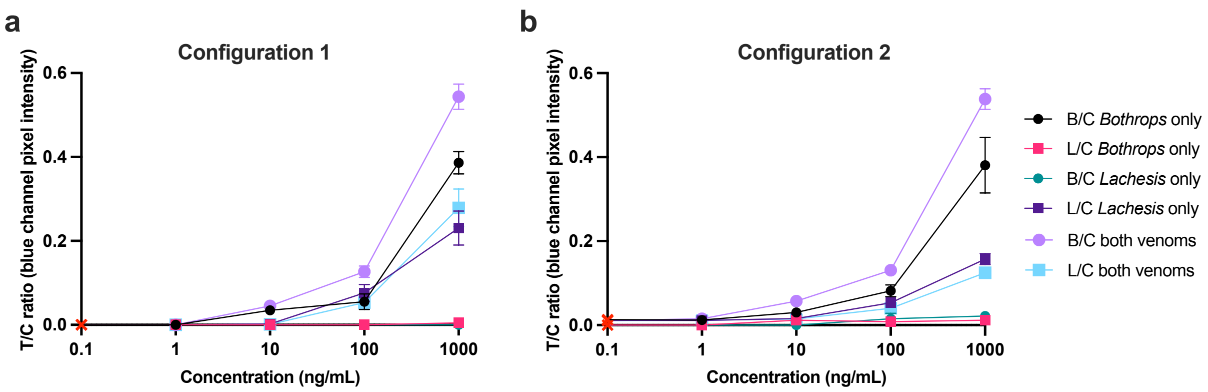

Fig. S5 Calibration curves comparing two configurations of multiplex LFAs tested in venom spiked running buffer; (a) configuration 1: control line, *Bothrops* line, *Lachesis* line; (b) configuration 2: control line, *Lachesis* line, *Bothrops* line, tested in increasing concentrations (1-1,000 ng/mL) of each individual venom (*B. atrox* or *L. muta*) or both venoms simultaneously. Curves were plotted as *Bothrops* test line and *Lachesis* test line signal intensities divided by their corresponding control line signal intensity (B/C and L/C) against concentration. Error bars represent the standard deviation (n=3), and the red cross represents the signal in blank running buffer.

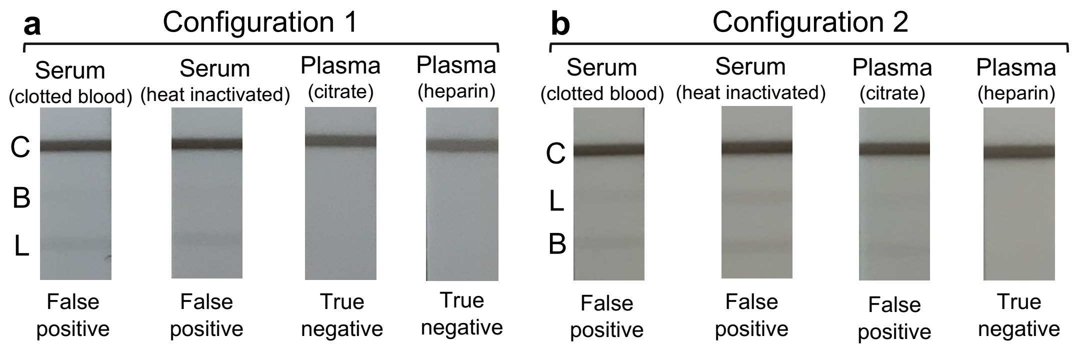

Fig. S6 Photos of the two multiplex LFA configurations tested in different blank serum and plasma matrices, using 10 µL matrix in 90 µL running buffer; (a) configuration 1 [Control line > *Bothrops* line > *Lachesis* line]; (b) configuration 2 [Control line > *Lachesis* line > *Bothrops* line].

Table S2 List of snake venoms used in this study

Genus	Species and subspecies	Geographical origin	Manufacturer/donor
Bothrops	*alternatus**†	Unspecified	Kentucky Reptile Zoo, cat# BAL
Bothrops	*asper**	Costa Rica, Ecuador	Latoxan, cat#L1209
Bothrops	*atrox**†	Brazil	Latoxan, cat#L1210A
Bothrops	*atrox**	Colombia	Kentucky Reptile Zoo, cat# BAt-C
Bothrops	*atrox**	Suriname	Kentucky Reptile Zoo, cat# BAt-S
Bothrops	*diporus**†	South America	Latoxan, cat# L1267
Bothrops	*jararaca**†	Brazil	Latoxan, cat# L1211
Bothrops	*jararacussu**†	Brazil	Latoxan, cat# L1256
Bothrops	*leucurus**†	Brazil	Latoxan, cat# L1255
Bothrops	*mattogrossensis**†	Brazil	Latoxan, cat# L1248
Bothrops	*moojeni**†	Brazil	Latoxan, cat# L1254
Bothrops	*neuwiedi**†	Brazil	Latoxan, cat# L1213
Bothrops	*pauloensis**†	Paraguay	Latoxan, cat# L1265
Crotalus	*adamanteus**	Unspecified	Kentucky Reptile Zoo, cat# EDB
Crotalus	*atrox**	Unspecified	Kentucky Reptile Zoo, cat# CAt
Crotalus	*durissus terrificus**†	Brazil	Latoxan, cat# L1261B
Crotalus	*horridus**	Unspecified	Kentucky Reptile Zoo, cat# CH
Crotalus	*scutulatus scutulatus**	Unspecified	Kentucky Reptile Zoo, cat# CSS-T
Crotalus	*simus**	Costa Rica	Latoxan, cat#L1260
Lachesis	*melanocephala**	Costa Rica	Prof. Bruno Lomonte
Lachesis	*muta**†	Suriname	Latoxan, cat# L1290B
Lachesis	*muta muta*†	Peru	Dr Alfonso Zavaleta & Dr Maria Salas

*Antibody binding to these venoms was tested in ELISAs. †Antibody binding to these venoms was tested in LFAs.

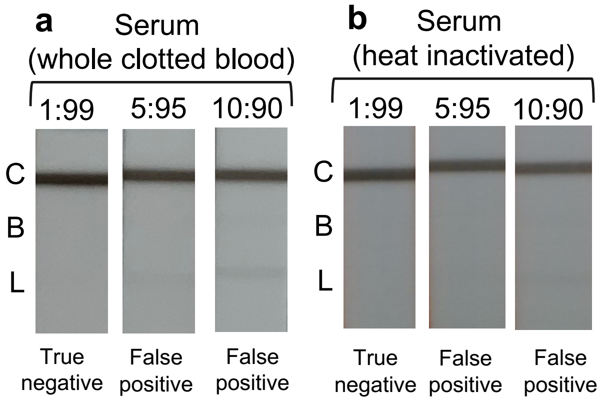

Fig. S7 Photos of the optimal multiplex LFA (configuration 1) tested in different blank serum-to-running buffer ratios; 1 µL serum in 99 µL running buffer, 5 µL serum in 95 µL running buffer, and 10 µL serum in 90 µL running buffer, resulting in three different dilutions factors (100x, 20x, and 10x) in (a) serum from clotted blood and (b) heat-inactivated serum. C denotes the control line, B denotes the *Bothrops* test line, and L denotes the *Lachesis* test line.

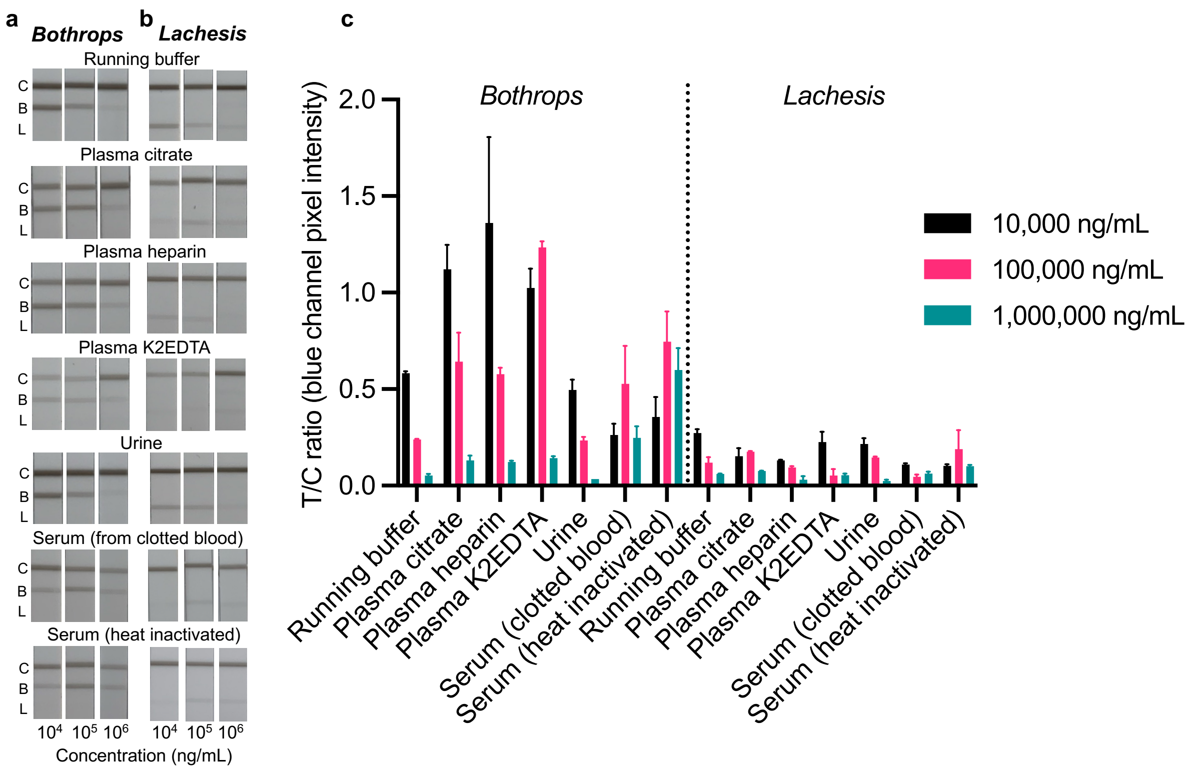

Fig. S8 Multiplex LFA tested at high antigen concentrations (10,000, 100,000, 1,000,000 ng/mL) in running buffer and six different biological matrices (plasma (citrate, heparin, K2EDTA), serum (from clotted blood, heat-inactivated); (a) photos of LFAs used to detect *B. atrox* or *L. muta* venom in spiked matrices; (b) bar chart showing the test line divided by control line (T/C) signals. Here, black represents a concentration of 10,000 ng/mL, pink represents 100,000 ng/mL, and teal represents 1,000,000 ng/mL.

Table S3 List of matrixes used in this study

Matrix	Anticoagulant or details	Identification	Donor details	Collection date	Company
Plasma	K2EDTA	PLSSKF2EDT25-MSOP	Male, O RhD pos, 35 y/o.	2020.03.14	Research Donors, UK
Plasma	Sodium citrate	PLSSKF2CIT25-FSXX	Female, O RhD pos, 46 y/o.	2020.07.31	Research Donors, UK
Plasma	Sodium heparin	PLSSKF2SHE25-MSOP	Male, O RhD pos. 27 y/o	2020.06.09	Research Donors, UK
Serum	Heat inactivated, from whole clotted blood	H5667	Male, AB	Not reported	Sigma Aldrich
Serum	From whole clotted blood	H6914	Male, AB	Not reported	Sigma Aldrich
Urine	-	991-03-P	Pooled $\geq$3 donors per pool	Not reported	Lee biosolutions

Table S4 Overview of experiments performed with the developed LFAs

Experiment	Biological matrix tested	Summary & test conditions
Optimization & selection of running buffer (RB)	Sera (from clotted blood & heat-inactivated), plasma (citrate & heparin)	*Bothrops* LFAs tested in RB + Tween-20 or Tergitol +/- NaCl; *Lachesis* LFAs tested in RB + Tween-20 or Tergitol + NaCl<br>90 µL RB: 10 µL blank matrix
LoDs of singleplex LFAs in RB	N/A	Singleplex LFAs tested in blank & venom-spiked RB (1-1,000 ng/mL)<br>90 µL RB: 10 µL spiked RB
Comparison of multiplex LFA configurations in RB	N/A	Both LFA configurations tested in blank & venom-spiked RB (1-1,000 ng/mL)<br>90 µL RB: 10 µL spiked RB
Cross reactivity testing	N/A	LFA tested in 13 different venoms spiked into RB (100 & 1000 ng/mL)<br>90 µL RB: 10 µL spiked RB
LoDs of LFA in spiked urine and plasma	Urine, plasma (citrate, heparin, & K2EDTA)	LFA tested in blank & venom-spiked urine & plasma matrices (1-1,000 ng/mL)<br>90 µL RB: 10 µL spiked matrix
Troubleshooting in serum	Sera (from clotted blood & heat-inactivated)	LFA tested in blank sera using different sample to RB ratios: (i) 99 µL RB: 1 µL blank sera; (ii) 95 µL RB: 5 µL blank sera; (iii) 90 µL RB: 10 µL blank sera
LoDs of LFA in spiked serum	Sera (from clotted blood & heat-inactivated)	LFA tested in blank & venom-spiked sera (1-1,000 ng/mL)<br>99 µL RB: 1 µL spiked sera
Matrix effects	Plasma (citrate, heparin, & K2EDTA), urine, sera (from clotted blood & heat-inactivated)	LFA tested in 20 repeat measurements in blank matrices: (i) 90 µL RB: 10 µL blank RB, plasma, or urine; (ii) 99 µL RB: 1 µL blank sera

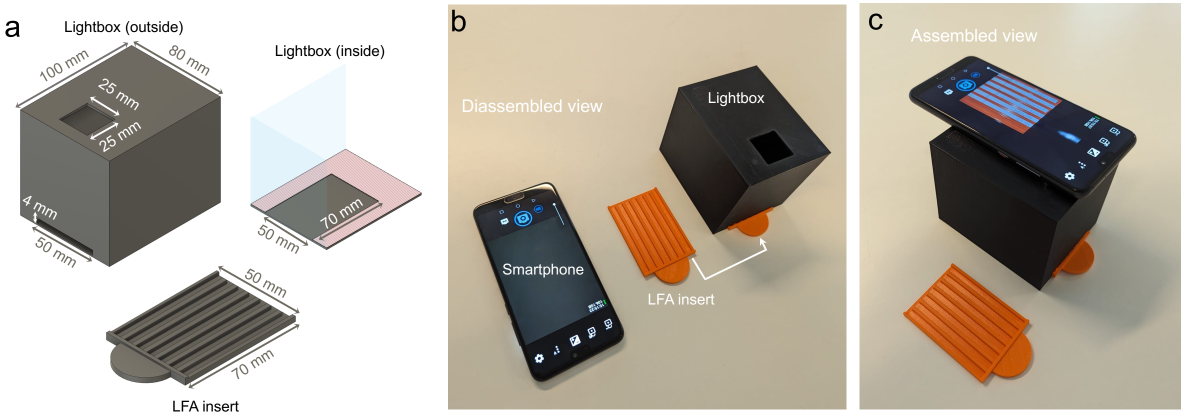

Fig. S9 3D printed platform for reading LFAs under controlled lighting conditions; (a) computer aided design (CAD) of the lightbox and LFA insert; (b) photo of the disassembled parts of the platform; (c) photo of the assembled platform with smartphone attached and recording of developed LFA strips.

Protocol S1: (Semi)quantitatively analyzing LFAs using ImageJ

1. Open photo of LFAs in ImageJ (a).
2. Split the photo into color channels [Image>Color>Split] and select the blue channel.
3. Use the line command (width of >35) to draw a line covering the strip (b).
4. Analyze the pixel intensity of the background, control line, and test line(s) [Analyze>Plot profile] (c).
5. Extract the plot values from the peak and list.
6. Copy extracted data to spreadsheet.
7. Plot data by subtracting the test and control peak readings from the background peak reading.
8. Divide the average test line value by the corresponding average control line value and plot this average T/C ratio against antigen concentration with error bars to represent the standard deviation between measurements (n=3).

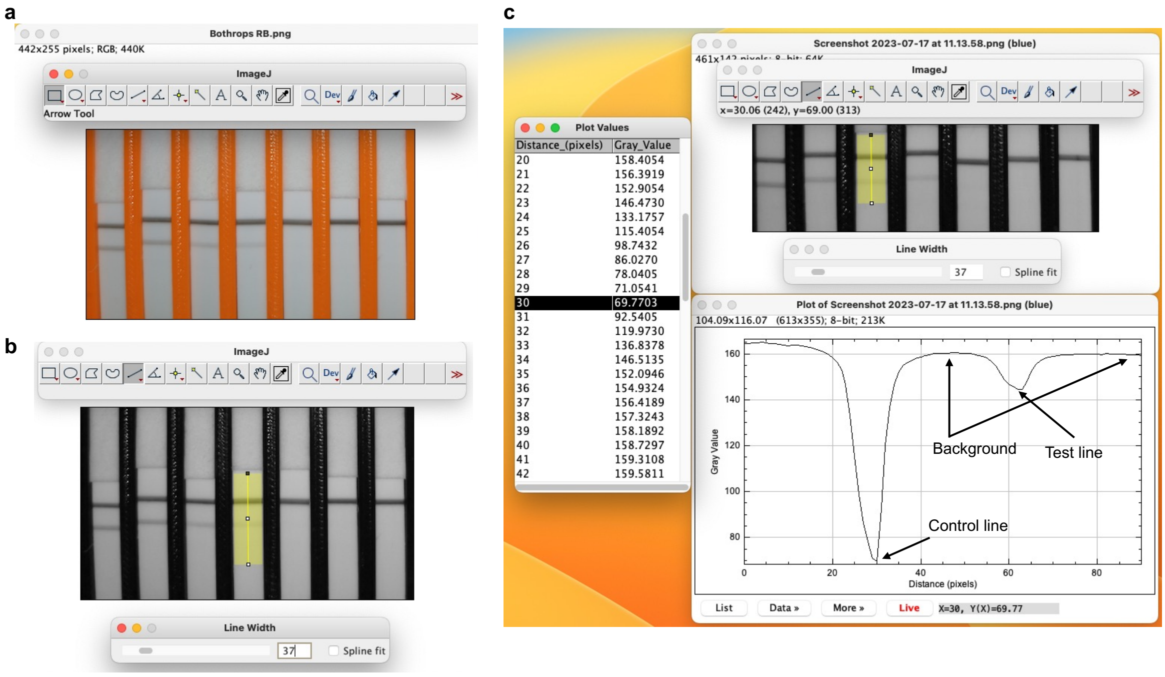

Supplement: Supplementary file 1 — Supplementary Information. [file 41598_2024_51971_MOESM1_ESM.pdf]
